# TNF-α Is Involved in the Abnormal Thymocyte Migration during Experimental *Trypanosoma cruzi* Infection and Favors the Export of Immature Cells

**DOI:** 10.1371/journal.pone.0034360

**Published:** 2012-03-26

**Authors:** Ana Rosa Pérez, Luiz Ricardo Berbert, Ailin Lepletier, Silvia Revelli, Oscar Bottasso, Suse Dayse Silva-Barbosa, Wilson Savino

**Affiliations:** 1 Faculty of Medical Sciences, Institute of Immunology, National University of Rosario, Rosario, Argentina; 2 Laboratory on Thymus Research, Oswaldo Cruz Institute, Oswaldo Cruz Foundation, Rio de Janeiro, Brazil; Federal University of São Paulo, Brazil

## Abstract

Previous studies revealed a significant production of inflammatory cytokines together with severe thymic atrophy and thymocyte migratory disturbances during experimental Chagas disease. Migratory activity of thymocytes and mature T cells seem to be finely tuned by cytokines, chemokines and extracellular matrix (ECM) components. Systemic TNF-α is enhanced during infection and appears to be crucial in the response against the parasite. However, it also seems to be involved in disease pathology, since it is implicated in the arrival of T cells to effector sites, including the myocardium. Herein, we analyzed the role of TNF-α in the migratory activity of thymocytes in *Trypanosoma cruzi* (*T. cruzi*) acutely-infected mice. We found increased expression and deposition of TNF-α in the thymus of infected animals compared to controls, accompanied by increased co-localization of fibronectin, a cell migration-related ECM molecule, whose contents in the thymus of infected mice is also augmented. *In-vivo* studies showed an enhanced export of thymocytes in *T. cruzi*-infected mice, as ascertained by intrathymic injection of FITC alone or in combination with TNF-α. The increase of immature CD4^+^CD8^+^ T cells in secondary lymphoid organs was even more clear-cut when TNF-α was co-injected with FITC. *Ex-vivo* transmigration assays also revealed higher number of migrating cells when TNF-α was added onto fibronectin lattices, with higher input of all thymocyte subsets, including immature CD4^+^CD8^+^. Infected animals also exhibit enhanced levels of expression of both mRNA TNF-α receptors in the CD4^+^CD8^+^ subpopulation. Our findings suggest that in *T. cruzi* acute infection, when TNF-α is complexed with fibronectin, it favours the altered migration of thymocytes, promoting the release of mature and immature T cells to different compartments of the immune system. Conceptually, this work reinforces the notion that thymocyte migration is a multivectorial biological event in health and disease, and that TNF-α is a further player in the process.

## Introduction

*Trypanosoma cruzi* is the etiologic agent of Chagas disease. Despite substantial progress over the last years in relation to the immunopathology of the disease, the role of the thymus in the course of infection and pathogenesis remains unclear. Severe thymic alterations were observed during experimental *Trypanosoma cruzi*-acute infection. Thymuses in infected mice develop a progressive atrophy, mainly due to a depletion of CD4^+^CD8^+^ double-positive thymocytes [1], [2]. Such CD4^+^CD8^+^ cell depletion seems to be linked to a severe stress-triggered apoptosis [3]. Nevertheless, other simultaneous mechanisms of cell loss may take place, as for example a decrease in the arrival of bone marrow-derived precursors and/or an increase in thymocyte export. Recent data confirmed an altered intrathymic migration of thymocytes along with an atypical presence of immature CD4^+^CD8^+^ cells bearing prohibited TCR Vβ segments in secondary lymphoid organs [4]–[6]. These functional abnormalities were accompanied by an increase in the deposition of extracellular matrix proteins (ECMs) in cortex and medulla of the thymic lobules, and by a rise in the expression of their receptors in thymocyte subpopulations and peripheral CD4^+^CD8^+^ cells [4]–[7]. Furthermore, the anomalous exit of CD4^+^CD8^+^ cells seems to be influenced by the interaction between fibronectin and the chemokine CXCL12 [5].

The role of tumor necrosis factor-alpha (TNF-α) in *T. cruzi* infection seems to be dual. Control of human and experimental *T. cruzi* infection is critically dependent on TNF-α activity but its overproduction is detrimental to the host and contributes to disease pathology [2], [8]–[11]. The contribution of TNF-α to the *T. cruzi*-triggered thymic atrophy is at least partly dependent on hypothalamus-pituitary-adrenal axis activation, which promoted intrathymic apoptosis of CD4^+^CD8^+^ cells by exposure to increased levels of adrenal glucocorticoid hormones [3], [12].

Processes related to thymocyte migration could be also mediated by TNF-α, but at present, data on a putative role of TNF-α modulating cell migration in the thymus remains lacking. Yet, this hypothesis seems plausible since in *T. cruzi*-infected mice, TNF-α modulates migratory responses, inducing chemokine expression at systemic level, probably down-regulating CCR5 expression [13], [14]. We previously showed that in *T. cruzi*-infected TNF-α receptor double knockout mice (p55^−/−^ plus p75^−/−^) inflammatory infiltrates in the myocardium are absent [3], suggesting that during *T. cruzi* infection migration of peripheral T cells is influenced by TNF-α.

Under normal conditions, intrathymic cell migration and thymocyte export are complex processes, regulated at least by ECM proteins such as fibronectin and laminin, as well as chemokines [15]–[17], sphingosine-1-phosphate [18], [19], and hormones [20]–[22]. Considering that TNF-α is capable of interacting with fibronectin, promoting T cell adhesion [23], it is conceivable that during the immune response induced by *T. cruzi* infection, systemic or intrathymic production of TNF-α could modulate thymocyte migration, either by itself or in combination with ECM molecules, particularly fibronectin.

Taking into account previous results showing disturbances of thymocyte migratory properties during *T. cruzi* infection in mice, we explored herein the role of TNF-α in intrathymic T cell migration, as well as thymocyte export to peripheral lymphoid organs.

## Materials and Methods

### 1. Mice and experimental infection

Male C57BL/6 mice, aging 6–8 weeks, were obtained from the animal facilities at Rosario Medical School and Oswaldo Cruz Foundation. All animal procedures were performed according to protocols for animal care and use, approved by each Institutional Ethical Committee (Fiocruz Ethics Committee, Comissão de Ética no Uso de Animais (CEUA-Fiocruz), Resolution N° P-0145-02; and Faculty of Medical Sciences from National University of Rosario, Bioethics and Biosecurity Committees, Resolution N°3740/2009). Trypomastigotes of the Tulahuén strain of *T. cruzi* (Clonotype II) were maintained by serial passages in suckling mice. Heparinized blood obtained from infected animals was diluted in saline and washed twice. Live parasites were counted using Neubauer chambers. Mice were infected subcutaneously with 100 viable trypomastigotes. To monitor the systemic repercussion of the acute disease, parasitaemia and the survival time was recorded following infection as described before [3].

### 2. Flow cytometry analysis of cell suspensions

Thymuses, subcutaneous lymph nodes and spleens were removed, minced, washed and resuspended in PBS containing fetal calf serum 5% (Gibco, California, USA). Spleen samples were further treated with NH_4_Cl for red cell lysis.

For immunostaining, 1.10^6^ cells were resuspended in flow buffer and incubated with a given specific monoclonal antibody for 30 minutes at 4°C in the dark (PE/anti-CD4, PercP/anti-CD8 or TC/anti-CD8 antibodies, BD Pharmingen, San Diego, USA). Once defined the lymphocyte gate, 30,000 events were acquired. Background staining values obtained with fluorochrome matched-conjugate isotype controls were subtracted.

For recent thymic emigrants (RTEs) determination (see below), cells from mice injected with physiological saline alone were used to define positive FITC labeling in the other groups.

In cell migration experiments, migrating cells were also labelled with the appropriate antibodies, fixed with paraformaldehyde 1% and analyzed by flow cytometry. Monoclonal antibodies used to stain cell surface molecules were: PE/anti-CD4, PercP/anti-CD4, PE/anti-CD8, PercP/anti-CD8, APC/anti-CD8, APC/anti-CD3 and PE/anti-TCR (BD Pharmingen). In all cases, dead cells were gated out on the basis of forward- and side-cell scatter. Fluorochrome-labeled isotype-matched negative controls for the specific antibodies were also used (BD Pharmingen). Acquisition of events was done using an FACSAria or FACScalibur flow cytometers (BD Pharmingen). Results were analyzed using the WinMDI 2.8 software (Joseph Trotter, CA, USA).

### 3. Determination of circulating and intrathymic TNF-α contents

Mice were bled by cardiac puncture at different days post-infection. Blood was centrifuged during 10 min at 1,500 rpm at −4°C. Serum was stored frozen at −20°C until analyzed. At the same time, thymuses were removed, weighted and homogenized in 2 volumes of 300 mmol/L sucrose with protease inhibitors (1 mM phenylmethylsulfonyl fluoride, 10 µg/ml leupeptin, and 1 µg/ml aprotinin). Homogenates were kept at −80°C until used. TNF-α concentrations were measured in blood and tissue samples by a specific ELISA kit, according to manufacturer's specifications (BD Pharmingen). Detection limit of the technique corresponds to 15.6 pg/ml.

In further experiments, intrathymic TNF-α was detected by means of immunoblotting. Lysates of thymuses were prepared in 4 volumes of 300 mmol/L sucrose with protease inhibitors: 1 mM phenylmethylsulfonyl fluoride, 10 µg/ml leupeptin, and 1 µg/ml aprotinin (Sigma Co, St. Louis, USA). Samples (20 µg of protein) were subjected to electrophoresis under reducing conditions on a 15% SDS-PAGE, and transferred onto nitrocellulose membranes. After blocking (5% non fat milk during 2 h at room temperature), blots were incubated overnight at 4°C with rat anti-mouse TNF-α monoclonal antibody (clone MP6-XT3). Membranes were then incubated in blocking buffer with anti-rat Ig G-peroxidase conjugate (1∶5000, Amersham Life Science, Buckinghamshire, UK) and bands were detected by enhanced chemiluminescence kit (Amersham). Autoradiographs were obtained by exposing PVDF membranes to Kodak XAR film, and the bands were quantitated by densitometry, using a Shimadzu CS-9000 apparatus (Kyoto, Japan).

### 4. In situ detection of TNF-α in the thymus

Thymuses were removed 14 days after infection, embedded in Tissue-Tek (Miles Inc., Elkhart, USA) and frozen in liquid nitrogen. Five 5 µm-thick cryostat sections were settled on poly-L-lysine (Sigma)-covered glass slides, acetone fixed and blocked with PBS-BSA 1%. Samples were submitted to specific antibodies as follows: 1∶10 rat anti-mouse TNF-α monoclonal antibody (1∶10, stock: 100 mg/mL, clone MP6-XT3 or rabbit anti-mouse fibronectin (Novotec, Saint-Martin La Garenne, France) for 1 hour at room temperature, washed and submitted to appropriate secondary antibody, 1∶400 goat anti-rabbit Alexa 488 and 1∶400 goat anti-rat Alexa 546 (Molecular Probes, Eugene, USA) respectively. Samples were analyzed by confocal microscopy using a LSM 510 Zeiss device (Germany) and the images obtained were subsequently analyzed using the Image J software (Bethesda, Maryland, USA).

### 5. Evaluation of recent thymic emigrants

Intrathymic injection of fluoresceinisothiocyanate (FITC) allows direct *in vivo* evaluation of RTEs. FITC efficiently and randomly labels thymocytes, enabling their subsequent identification in the peripheral lymph organs. Intrathymic FITC injections were performed as described by Scollay et al [24] with minor modifications. Briefly, animals were anesthetized (ketamine-100 mg/kg/xylaxine-2 mg/kg) and then the chest was opened to expose the thymus. A Hamilton syringe was used to inject 12.5 µL/lobe of a suspension containing TNF-α plus FITC or FITC alone. Recombinant murine TNF-α (PeproTech, Mexico City, Mexico) was diluted up to 200 pg/mL in 500 mg/mL FITC (Sigma-Aldrich, St. Louis, USA). FITC suspension was prepared from a saturated stock diluted in physiological saline. Control group was injected with saline alone. After intrathymic injection, the skin incision was closed and mice were allowed to recover under a heat source. To evaluate RTEs, animals were sacrificed 24 post-injection, and thymuses, spleen as well as axillary and inguinal subcutaneous lymph nodes were removed. Lymphoid cells from each organ were suspended in PBS solution containing 5% fetal calf serum; being then labeled with the appropriate antibodies for monitoring FITC^+^CD4^+^CD8^+^ cells by flow cytometry. Only animals with more than 50% of FITC^+^ thymocytes were used for subsequent analysis. Peripheral cells were considered RTEs when FITC^+^ cells could be distinguished from, comparing with animals that only received intrathymic injection of saline. Data were evaluated as absolute numbers of FITC^+^ cells, and also through an index enabling to estimate the overall rate of emigration (ORE), adjusted to the absolute number of FITC^+^CD4^+^CD8^+^ cells at the periphery, in relation to corresponding secondary lymphoid organ cellularity and the percentage of labelled cells observed in the thymus.
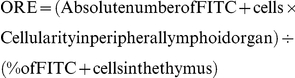



### 6. Cell migration assay

We evaluated thymocyte migratory response to TNF-α adhered to fibronectin. Briefly, inserts of 5 µm-pore size of transwell plates (Corning Costar, Cambridge, USA) were coated with 10 µg/mL of human fibronectin or 10 µg/ml BSA (Sigma) for 1 h at 37°C and then unspecific binding sites were blocked with BSA 10 µg/mL. Various concentrations of recombinant murine TNF-α (25 or 250 pg/mL) were diluted in 70 µL added to upper chamber, allowing adhesion to fibronectin (or BSA) during 15 min at 37°C. Unbound TNF-α was then removed by washing before adding thymic cells. Thymocytes were obtained at 14 days post-infection or from non-infected counterparts. Cells (2.5 10^6^ thymocytes in 100 µL of RPMI-1% BSA) were then added in the upper chambers. Migration medium was serum free, so that to avoid serum-derived fibronectin or other soluble cell migration stimuli. After 3 hours of incubation at 37°C in a 5% CO_2_ humidified atmosphere, migration was defined by counting the cells that migrated to the lower chambers containing migration medium alone (RPMI-1% BSA). Migrating cells were ultimately counted, labelled with appropriate antibodies and analyzed by flow cytometry. Differences in intrinsic cytokinesis between displayed thymocytes from infected or noninfected mice driven by TNF-α or fibronectin (alone or in combination) were expressed after subtracting the value recorded in wells containing BSA alone. The percentage of each subset was used together with the total cell counting to calculate absolute numbers of each lymphocyte subset from each lymphoid organ. The results are presented in terms of total thymocyte migration as well as the percentages of input seen in each CD4/CD8-defined subpopulation, using the following formula:
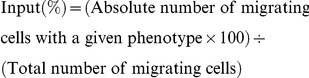



### 7. Expression of TNF-receptors in CD4/CD8-defined thymocyte subpopulations

Expression of genes coding for both TNF-receptors (TNF-R1 and TNF-R2) was evaluated by real-time PCR. In each experimental condition, thymocytes subsets were FACS isolated from three pooled thymuses stained with anti-CD4 Percp and anti-CD8 APC antibodies, in a MoFlocell sorter (DakoCytomation, Fort Collins, CO). All subsets were enriched to a purity of greater than 98%. After sorting, RNA was extracted using a commercially available kit (RNA easy minikit, Qiagen, Courtaboeuf, France). Total RNA concentrations were estimated by A_260_ measurement. First strand cDNA synthesis was prepared with 0.5 µg total RNA, random hexamer primer, and Superscript III reverse transcriptase (Invitrogen, Carlsbad, CA). For qPCR we used approximately 100 ng of cDNA for each sample and SYBR Green Master Mix 2 (Applied Biosystems, California, USA). cDNA was amplified using specific murine primer sequences: 5′-TCAAAGAGGAGAAGGCTGG-3′ (FP) and 5′-CACCAC AGCATACAGAATCG-3′ (RP) for TNF-R1; (5′ TGTAGCATCCTGGCTATTCC-3′ (FP) and 5′-ATGAAGCAGTTCACCAGTCC-3′ (RP) for TNF-R2 and 5′-GCTACAGCTTCACCACCACAG-3′ (FP) and 5′-GGTCTTTACGGATGTCAACGTC-3′ (reverse) for ACTB (β-actin). All reactions were performed in triplicate. After 45 cycles of amplification, TNF-R1 and TNF-R2 expression in each cell subset was normalized to the housekeeping gene ACTB (2^−ΔCt^×1,000) [25], subsequent to the following primer efficiency analysis.

### 8. Statistical analyses

Differences in quantitative measurements were assessed by the Kruskall-Wallis non parametric analysis of variance and Mann-Whitney U test. Correlations were evaluated by Pearson test. Results were expressed as mean ± standard error (SE) unless otherwise indicated. The GraphPad Instat 4.0 software (GraphPad, California, USA) was applied for statistical analyses, and differences were considered significant when *p* value was ≤0.05.

## Results

### Thymic atrophy in *T. cruzi* acutely-infected mice parallels enhanced circulating and intrathymic contents of TNF-α

**Figure 1:**
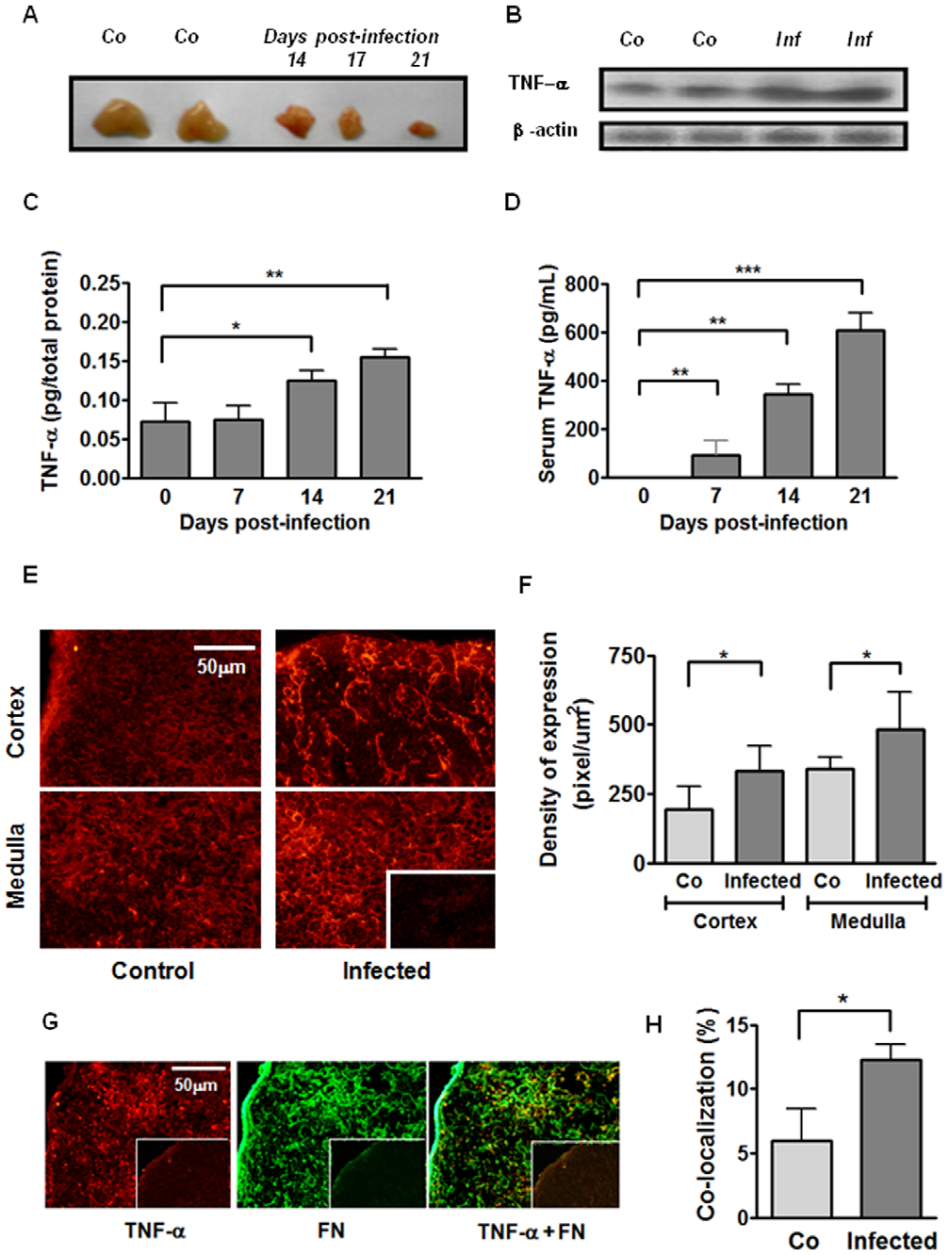
Enhanced circulating and intrathymic contents of TNF-α parallel the thymic atrophy in *T. cruzi* acutely-infected mice. **A**) Representative picture showing two thymuses from healthy control mice (Co) and the progressive thymic atrophy after 14, 17 and 21 days of acute infection; **B**) TNF-α protein detection in thymus homogenates by western blot. Representative photomicrographs showing TNF-α and β-actin expression in two control mice (*Co*) and in two infected mice after 21 days post-infection (*Inf*); **C**) TNF-α concentrations in thymic homogenates detected by ELISA at different days after infection and normalized to total protein contents. Bars represent the mean ± SEM of three pools/day); **D**) Systemic concentration of TNF-α along infection evaluated by ELISA. (n = 5–8 mice/day); **E**) Confocal microscopy showing an enhancement in TNF-α contents both in cortex and medulla of the thymic lobules, after 14 days of infection. Original magnification 400×. Small box is a representative control staining in which an unrelated primary antibody was applied; **F**) Graphs correspond to relative quantification analysis of TNF-α deposition in both cortex and medulla from 3–5 microscopic fields of thymuses from control (n = 5) or 14 days-infected animals (n = 5); **G**) Representative immunofluorescence staining showing thymuses of infected mice: TNF-α expression (in red, left picture); fibronectin (FN) deposition (in green, middle picture); co-localization of TNF-α and FN (right picture). Small boxes correspond to negative control where immune reaction was controlled by using unrelated primary antibodies; **H**) Bars correspond to the percentages of TNF-α plus FN co-localization in thymuses from control mice compared with infected counterparts. Significant differences are indicated as *p<0.05, **p<0.01, ***p<0.001.

A progressive thymic atrophy characterized by a diminution in the organ size (Fig. 1a) linked to a marked depletion of CD4^+^CD8^+^ thymocytes (data not shown) was seen along with infection in C57BL/6 infected mice, thus confirming previous results. These alterations occurred in parallel with a systemic and intrathymic increase in TNF-α contents. TNF-α rising became evident 14 days post-infection in both thymus extracts and serum (Fig. 1b–c–d). Actually, there was a strong correlation between intrathymic and systemic concentrations of TNF-α (p<0.01). Additionally, we found that the higher intrathymic TNF-α contents inversely correlated with the thymic relative weight and the relative numbers of CD4^+^CD8^+^ cells, thymic parameters that declined along infection (Table 1). Confocal microscopy confirmed a more intense deposition of TNF-α both in cortex and medulla of thymic lobules after infection (Fig. 1e–f).

**Table 1:** Correlation analyses for TNF-α thymic concentrations and atrophy parameters in *T. cruzi* acutely infected mice.

Correlation between	TNF-α thymic contents (pg/50 mg tissue)	Rho	n
Serum TNF-α (pg/ml)	p<0.015	0.890	18
RTW	p<0.001	−0,693	18
% DP	p<0.001	−0.854	18

RTW = relative thymic weight (thymic weight/corporal weight); DP = CD4^+^CD8^+^ double positive cells; rho = Spearman correlation coefficient, n = number of XY pairs.

Since an abnormal intrathymic microenvironment could play a role in the atypical thymocyte exportation and considering previous report showing that in acutely-infected BALB*l*c mice the anomalous migration was linked to a higher deposition and expression of fibronectin together with enhanced co-localization of chemokines (Mendes-da-Cruz et al, 2006), we next evaluated whether thymic contents of TNF-α co-localized with fibronectin. Confocal microscopy data showed that co-localization of TNF-α and fibronectin was increased after infection (Fig. 1g–h).

### TNF-α modulates thymocyte export *in vivo*

**Figure 2:**
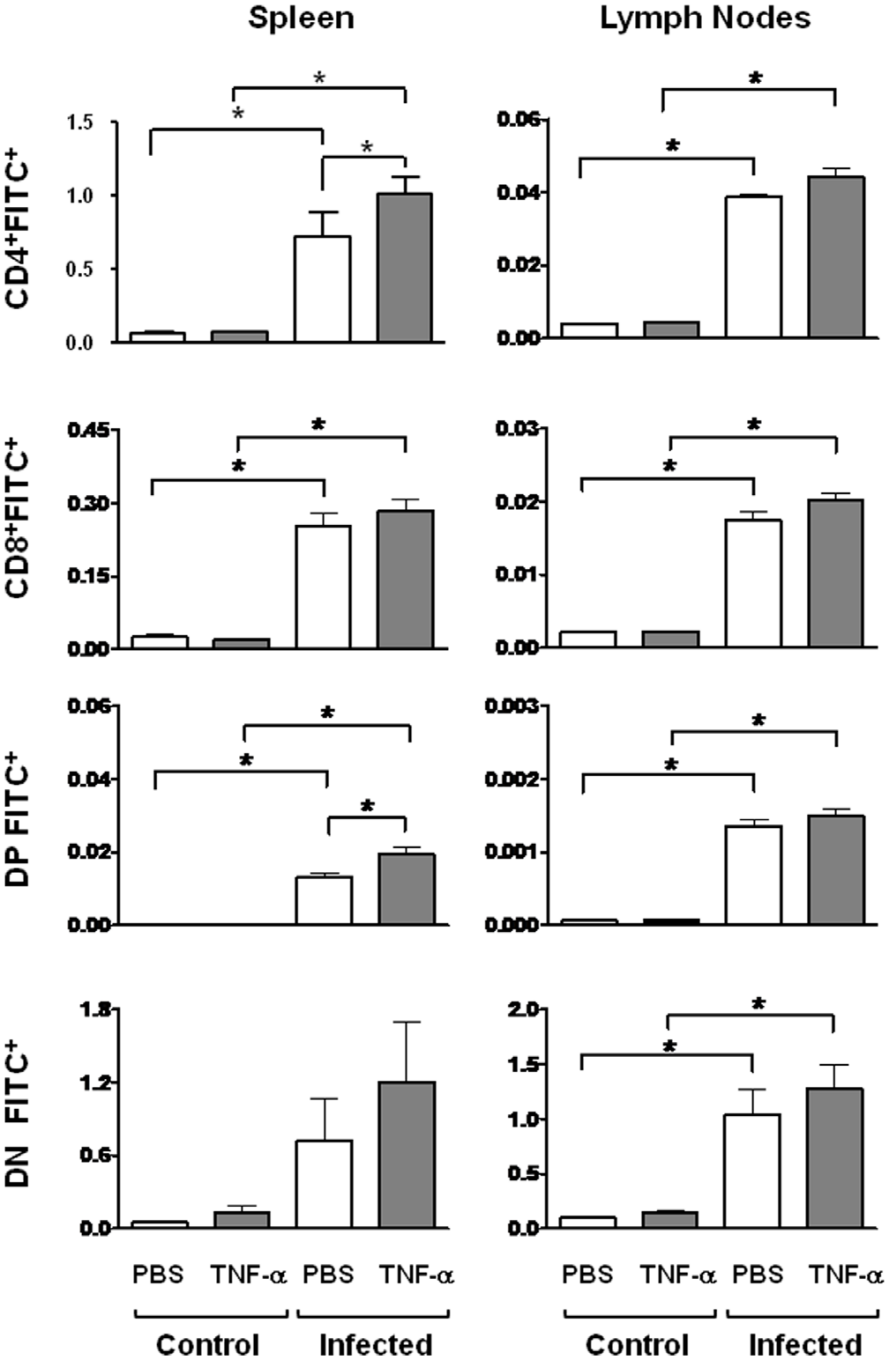
TNF-α enhances export of CD4^+^CD8^+^ thymocytes. Control and 14 days-infected animals were intrathymically injected with FITC dissolved in PBS with or without TNF-α. Twenty-four hours later, recent thymic emigrants (RTEs) were recognized as FITC^+^/CD4^+^/CD8^+^ cells in secondary lymphoid organs (spleen and subcutaneous lymph nodes) by flow cytometry. Empty bars represent the absolute number of FITC^+^ cells (×10^3^) from PBS injected mice and black bars represent the absolute number of FITC^+^ cells from TNF-α injected animals. Results show that after infection, the numbers of RTEs are clearly enhanced, independently of whether intrathymic injection of TNF-α was applied or not. When considering CD4/CD8-defined subpopulations, we found that intrathymic inoculation of TNF-α further promoted a significant enhancement in the numbers of FITC^+^CD4^+^ and FITC^+^CD4^+^CD8^+^ cell in the spleen, but not in lymph nodes. Data depict a representative experiment of two similar ones; each experiment being done with 5–6 animals/group. Statistically significant differences are indicated as *p<0.05.

To better approach the possibility that TNF-α plays a role upon migratory properties of thymocytes during acute *T. cruzi* infection, we investigated thymocyte export *in vivo*, by means of intrathymic FITC injection, alone or in combination with the cytokine. Twenty-four hours following intrathymic injection, thymuses, axillary and inguinal subcutaneous lymph nodes (SLN), as well as spleens, were removed and the FITC^+^ CD4/CD8-defined T cell subsets were ascertained by cytofluorometry (Fig. 2). Fourteen days after infection, there was a significant increase in absolute numbers of FITC^+^ cells in peripheral lymphoid organs, as compared to noninfected counterparts. Such an increase could be seen in infected mice injected with FITC alone or with FITC plus TNF-α. When the thymus was injected with FITC alone, absolute numbers of FICT^+^CD4^+^ and FITC^+^CD8^+^ simple positive cells from *T. cruzi*-infected mice were increased in both spleen and SLN, compared with noninfected controls. In addition, FITC^+^CD4^+^CD8^+^ cells were detected in secondary lymphoid organs from infected mice. Moreover, in infected animals, intrathymic injection of TNF-α plus FITC revealed an increase in the numbers of FITC^+^CD4^+^ and FITC^+^ CD4^+^CD8^+^ in the spleen, when compared with the injection of FITC in the absence of the cytokine. Differences in the absolute numbers of FITC^+^CD4^+^ or FITC^+^ CD4^+^CD8^+^ populations from infected animals were not detected in the SLN. Also, the amounts of FITC^+^CD8^+^ or FITC^+^CD4^−^CD8^−^ populations remained unchanged following injection with TNF-α plus FITC, compared with FITC alone.

Taking into account that the absolute numbers of FITC^+^ cells in the periphery depends on the degree of labelling of the thymus, we estimated the overall rate of emigration (ORE) by adjusting the absolute number of FITC^+^CD4^+^CD8^+^ cells in the periphery in relation to the cellularity of a given peripheral lymphoid organ and the percentage of labelled observed in thymus. As seen in Table 2, the ORE was affected by the infection, increasing several times their baseline values, independently whether the thymus were injected or not with TNF-α Interestingly, TNF-α plus FITC injection caused a marked increase in the ORE of FITC^+^CD4^+^CD8^+^ cells compared with FITC alone in both secondary lymphoid organs of infected animals. We did not notice differences in values from FITC^+^CD4^+^ RTEs in the spleen after TNF-α intrathymic injection, but, evaluation of ORE values suggest a TNF-α-driven enhancement of FITC^+^CD4^+^ and FITC^+^CD8^+^ cells in the lymph nodes from infected mice.

**Table 2:** Overall rate of emigration (ORE) after intrathymic inoculation.

*ORE*	Spleen		Subcutaneous Lymph Nodes	
	FITC alone	TNF-α+FITC	FITC alone	TNF-α+FITC
***Total RTEs***				
Control	3.3±0.5	8.0±2.0	3.0±0.8	5.0±0.10
*T. cruzi*	204.7±34.7^(**)^	314.6±79.6^(**)^	99.5 ±31.6^(**)^	170.8±31.7^(**)^
***CD4^+^ cells***				
Control	1.6±0.4	2.4±1.4	0.03±0.01	0.05±0.01
*T. cruzi*	88.4±32.0 ^(**)^	137.0±8.0 ^(**)^	1.15±0.25 ^(**)^	1.70±0.28 ^(**); (ΔΔ)^
**CD8^+^** ***cells***				
Control	0.8±0.3	0.7±0.1	2.1±0.1	2.2±0.4
*T. cruzi*	28.6±8.9 ^(**)^	37.0±4.9 ^(**)^	57.0±18.0 ^(**)^	88.7±11.8 ^(**); (Δ)^
***CD4^+^CD8^+^ cells***				
Control	0.1±0.0	0.6±0.3	0.0±0.0	0.4±0.3
*T. cruzi*	11.8±4.5	25.0±3.9^(**); (ΔΔ)^	1.0±0.3 ^(**)^	1.80±0.02 ^(**); (Δ)^
***CD4^−^CD8^−^ cells***				
Control	1.3 0±0.1	4.9±2.1	2.8±0.9	4.8±0.1
*T. cruzi*	72.0±45.0	158.2±75.0	94.5±33.8 ^(**)^	161.3±36.2 ^(**)^

Control and 14 days-infected animals were injected intrathymically with FITC or FITC plus TNF-α. Twenty-four hours after, thymuses, spleens and subcutaneous lymph nodes were screened for FITC^+^ CD4/CD8-defined cell subsets and these data were adjusted in relation to the cellularity of each peripheral lymphoid organ and the percentage of thymus labelling, using the following formula: Overall rate of emigration (ORE) = (Absolute numbers of FITC^+^ cells×cellularity in the peripheral lymphoid organ)/(% of FITC^+^ cells in thymus). We verified an enlarged ORE values in infected animals, independently of whether they were inoculated or not with TNF-α. Data indicate that TNF-α promotes the output of immature DP^+^ cells to both spleen and subcutaneous lymph nodes. Absolute numbers of FITC^+^ cells, CD4^+^, CD8^+^ and DN cells was expressed on 1.10^4^ cells, while DP^+^ cells was expressed on 1.10^3^ cells. Cellularity of each peripheral lymphoid organ was expressed in 1.10^6^ cells. Values are mean ± SEM of 5–6 mice/group (one representative experiment of two independent series). Difference between Control *versus T. cruzi* group = ^(^*^)^p<0.05; ^(^**^)^p<0.02. Differences between TNF-α treated *versus* not treated group = ^(Δ)^p<0.05; ^(ΔΔ)^p<0.02.

### TNF-α modulates fibronectin-driven *ex-vivo* migratory response of thymocytes

TNF-α lacks chemotactic activity by itself when placed in its soluble form in the bottom well of transwell cell migration chambers. Accordingly, migratory activity of thymocytes through membranes treated with BSA plus TNF-α was similar to that seen on BSA alone (data not shown).

**Figure 3:**
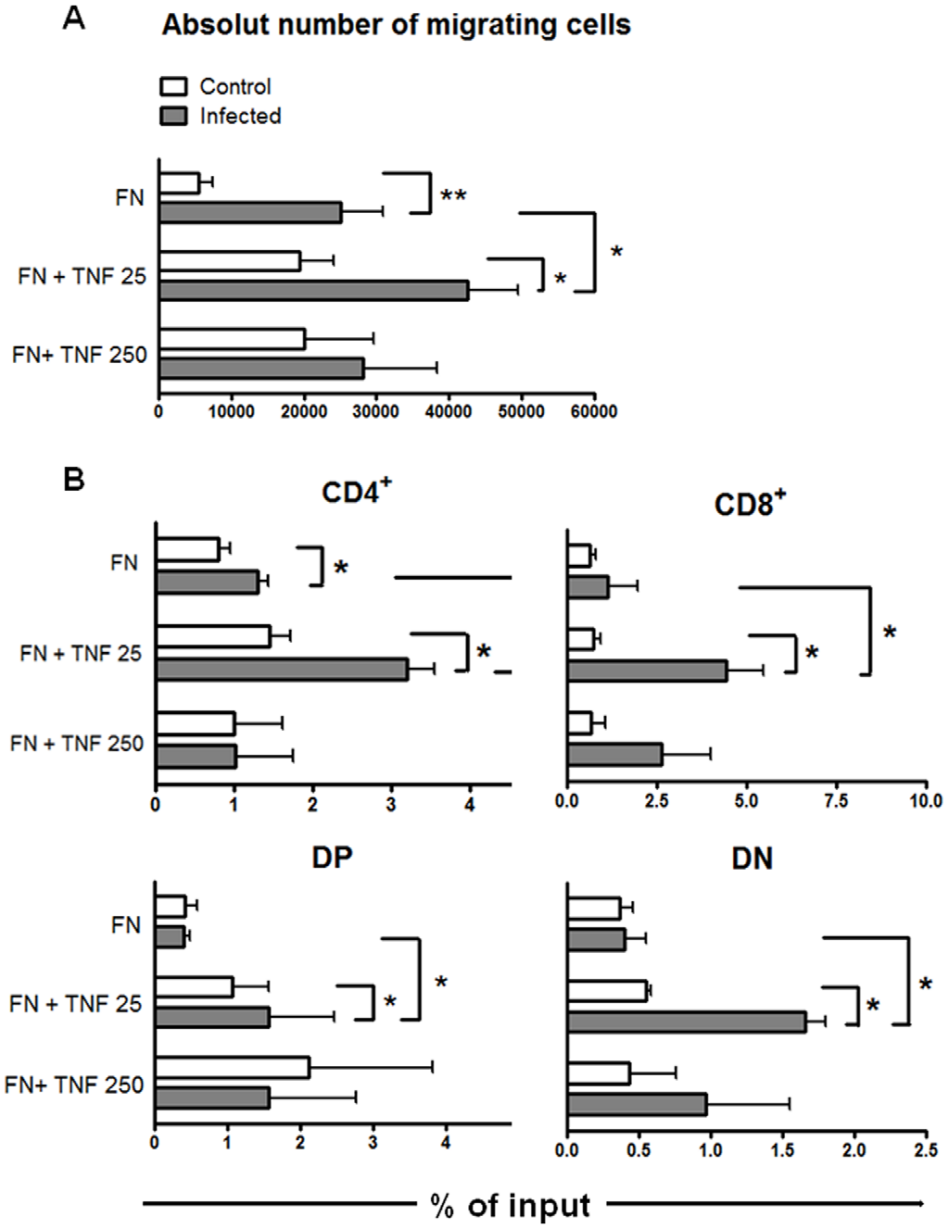
TNF-α enhances *in vitro* fibronectin-driven migration of thymocytes from *T. cruzi* infected mice. **A**) Thymocytes from control or infected animals were allowed to migrate in transwell chambers coated with fibronectin (FN) alone or FN plus TNF-α. Fibronectin-induced haptotactic response of thymocytes from infected animals was enhanced compared with controls in presence of TNF-α at the concentration of 25 pg/mL. Results derive from four experiments for TNF-25 pg/mL and three for TNF-250 pg/mL (each one obtained by pools of at least 3–4 animals by group) and correspond to specific migration after subtracting absolute cell numbers obtained in each well coated only with BSA. Statistically significant differences are indicated as p<0.05. **B**) Specific migration of thymocyte subpopulations was expressed as percentage of input (see Material and Methods session). We observed an enhanced migratory response of CD4^+^, CD8^+^, DP and DN cells when TNF-α was applied at 25 pg/mL, as compared with FN alone in infected animals. Results derived from four experiments, each one obtained by pools of at least 3–4 animals by group. Data correspond to specific migration after subtracting absolute cell numbers obtained in each well coated only with BSA. Statistically significant differences are indicated as *p<0.05 and **p<0.01.

To evaluate a haptotactic capacity of TNF-α, the recombinant cytokine was placed in the upper chamber 15 min before adding thymocytes. This procedure allows TNF-α to be deposited on the pre-formed fibronectin lattice. As previously reported [5], there is a higher fibronectin-driven migratory capacity of thymocytes from infected animals than the corresponding controls. Herein, we also showed that thymocytes from infected animals migrate more through fibronectin plus TNF-α than fibronectin alone (Fig. 3a). Thymocytes from infected animals also migrated more on fibronectin when the cytokine was added at a low concentration, namely 25 pg/mL. Nevertheless, compared with fibronectin alone, we found no statistically differences in the absolute numbers of migrating cells when 250 pg/mL TNF-α were added onto the fibronectin lattice (for comparison of fibronectin *versus* fibronectin plus TNF 250 pg/mL in both control and infected groups, we found *p* = 0.06).

For a more precise interpretation of these data, we evaluated the specific migration of CD4/CD8-defined thymocyte subsets (expressed as percentage of input for each subset), taking into account that the relative amounts of distinct CD4/CD8-defined thymocyte subpopulations changed after infection. We noticed that CD4^+^ cells from infected mice have a significantly higher migratory response than non-infected animals when they migrated through fibronectin alone, or through fibronectin plus 25 pg/mL TNF-α coated wells (group referred as FN+TNF25) (Fig. 3b). Furthermore, when this TNF-α concentration was added onto fibronectin, migratory responses of CD4^+^, CD8^+^ as well as CD4^−^CD8^−^ and CD4^+^CD8^+^ cells from infected animals were significantly enhanced compared with fibronectin alone, suggesting a co-stimulatory effect of the cytokine upon the fibronectin-driven migration of these thymocyte subsets. Interestingly, no differences were seen in the percentages of input, when thymocytes from control animals migrated through fibronectin plus TNF-α (independently of the concentration applied) compared with fibronectin alone; suggesting that TNF-α interacts with fibronectin mobilizing thymocytes from infected but not from control animals.

### Gene expression of TNF receptors in thymocytes from *T. cruzi* infected mice

**Figure 4:**
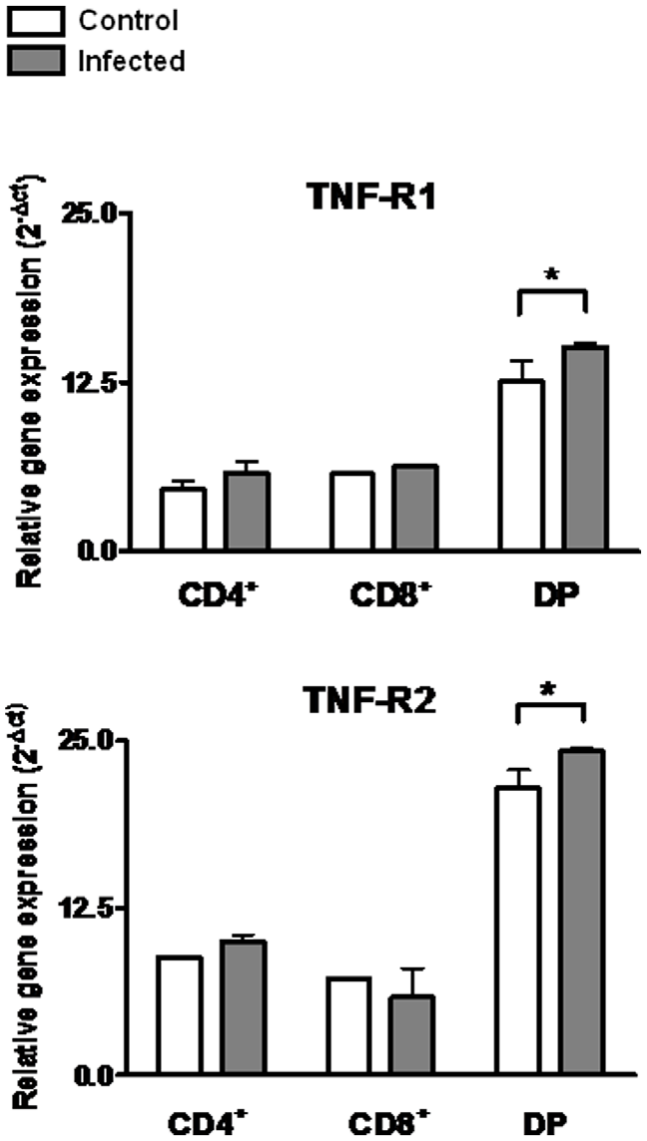
Expression of TNF-R1 and TNF-R2 en CD4^+^, CD8^+^ and CD4^+^CD8^+^ (DP) thymocytes. DP and SP T cells were purified from thymuses of control and acutely infected mice (14 days post-infection) by cell sorting using flow cytometry. To quantitatively evaluate TNFR transcripts, total mRNA samples from highly purified fresh DP, CD4+ and CD8+ T cells were processed for quantitative RT-PCR. The relative gene expression of mRNA of TNF-R1 and TNF-R2 were enhanced in DP thymocytes from infected animals compared with controls. Results are presented as relative gene expression, where mRNA levels were calculated using the equation 2^−ΔCt^ (difference in Ct between β-actin and the target gene). Data depict a representative experiment. Statistically significant differences are indicated as *p<0.05.

The enhanced migratory response of thymocyte subpopulations in *T. cruzi* infected mice might be related to the enhanced expression of integrin-type fibronectin receptors VLA-4 and VLA-5 [4], as well as the TNF-α receptors. We further compared the changes in the levels of TNF-R1 and TNF-R2 gene transcripts, ascertained herein by p55 and P75 gene expression respectively, in each CD4/CD8-defined subpopulation obtained from thymuses of control or infected animals (14 days post-infection). Based on the real-time RT-PCR analysis, a slight, yet significant induction of both receptors was detected in DP cells of *T. cruzi* infected mice. By contrast, TNF-R1 or TNF-R2 mRNA were similar in CD4^+^ or CD8^+^ subpopulations, independently of whether they derived from infected or control animals. Interestingly, in both conditions, the amounts of TNF-R1 and –R2 transcripts on the DP subset seemed greater than in SP cells, with a higher expression of the isoform 2 of this receptor (Fig. 4).

## Discussion

Thymic atrophy occurs in a variety of infectious diseases, including, among others, AIDS, malaria, syphilis and *T. cruzi* infection [26]. Such atrophy derives from massive thymocyte depletion, and results in changes in thymocyte export with consequences upon the peripheral T-cell pool and the corresponding T cell repertoire. Previous data strongly indicate that the thymic involution during *T. cruzi* infection disrupts the homeostasis of the organ, leading to an aberrant output of developing T cells, which likely bypassed intrathymic negative selection events [4], [5], [27], and might be involved in the generation of autoimmunity. Yet, a definitive link between thymic functional abnormalities and the autoimmune events occurring in Chagas disease remains to be demonstrated.

Most of the information concerning the role of TNF-α is related to induction of apoptosis [28]–[30]. However, our previous data obtained in TNR-receptor double knockout mice revealed that TNF-α is not required to induce thymocyte apoptosis during acute *T. cruzi* infection. Nevertheless, the putative role of TNF-α in thymocyte migration has not been investigated. Herein, we examined the characteristics of thymic atrophy and thymic migratory events in *T. cruzi* acutely infected animals, in relation to the systemic and the intrathymic contents of TNF-α.

The results described above on traffic abnormalities in the more immature thymocyte subpopulations, mainly CD4^+^CD8^+^ cells, but also CD4^−^CD8^−^, cells unravel some relationship with an enhancement in the contents of TNF-α complexed with fibronectin, accompanied by the upregulation of the corresponding receptors.

We first showed that intrathymic and systemic contents of TNF-α were enhanced in acute *T. cruzi* infection. In this respect, it is noteworthy that chronically-infected patients also exhibit augmented TNF-α serum levels [31]. In this respect, it is plausible to conceive that sustained high levels of the cytokine remain from the acute to the chronic phase during the evolution of Chagas disease. Interestingly, during *T. cruzi* infection, an increased TNF-α expression was also observed in the thymus of offsprings and foetuses from *T. cruzi*-chronically infected mothers [32] as well as thymuses from animals treated with trans-sialidase, a *T. cruzi* virulence factor [33]. Furthermore, the decline in TNF-α levels, as a result of LPS desensitization, ameliorates the loss of cortical CD4^+^CD8^+^ cells during acute *T. cruzi* infection [10].

Besides the increase in the intrathymic contents of TNF-α and fibronectin in infected animals, we found enhanced co-localization of these molecules within the organ. This finding is in line with studies by Alon and co-workers [23], showing that when TNF-α is expressed in high amounts in inflamed tissues it forms complexes with fibronectin.

This TNF-α/fibronectin interaction seems to have functional implications in T cell migration during *T. cruzi* infection, since low doses of TNF-α pre-bound to fibronectin, induced an increase in the export of mature CD4^+^ and CD8^+^ as well as CD4^−^CD8^−^ immature thymocyte subsets. Furthermore, *in vivo* intrathymic injection of TNF-α resulted in an increase in the numbers of RTEs, including immature double-positive cells, thus suggesting an abnormal fluctuation in thymocyte export, which could be link to the autoimmune events seen in chagasic mice and humans.

Other possibility to explain these data is that *in vivo* TNF-α binding to fibronectin favours in thymocytes, the integration with other cell migration-related signals, such as chemokines, for example, enabling them to tune their migratory activity in a given microenvironment, which is clearly disorganized during acute infection. Future studies on the combined effects of TNF-α with other fibronectin-binding chemokine(s), as CXCL12, should be informative.

Our study is at variance with previous observations, in which TNF-α was proposed as a stop signal for leukocyte migration [34]. Most likely however, the differences in the procedures applied for studying cell migration and the experimental conditions used in these assays account for such discrepancy. For example, we used thymocytes and not naïve peripheral T cells for functional assays.

A relevant aspect derived from our results is that, both *in vivo* and in *vitro*, immature CD4^−^CD8^−^ as well as CD4^+^CD8^+^ cells displayed a higher migratory response to fibronectin in the presence of TNF-α. These results not only confirm higher fibronectin-driven immature cell migration during the acute phase of infection [4], [5], but also place TNF-α as co-stimulatory moiety for migratory responses triggered by the ECM protein.

We have previously proposed that thymocyte migration (both in health and disease) is a multivectorial event, from the action of various ligand/receptor interactions; each one corresponding to a single migration vector [35]. The results presented herein show that intrathymic T cell migration is still more complex, since the TNF-α mediated interactions, although not being able to induce thymocyte migration *per se*, enhance fibronectin-triggered migratory response of these cells. In acutely-infected mice, the ultimate migration patterns reveal the abnormal release of immature CD4^+^CD8^+^ lymphocytes towards secondary lymphoid organs. Previous work strongly indicates that part of these immature cells released from the thymus in *T. cruzi* infected animals is potentially autoimmune, since they have bypassed the classic intrathymic negative selection [5], [40]. More recently, we showed that such a skewed negative selection is not due to an intrinsic defect of the thymic microenvironmental machinery, since thymic epithelial cell function seems normal in terms of AIRE (autoimmune regulator) and tissues restricted antigen gene expression [27]. Accordingly, it is conceivable that the release of immature cells from the thymus into the periphery is rather a cell migration-related disturbance.

In any case, the release of potentially autoimmune T cells may result in the development of autoimmunity, even in adult individuals. Importantly, recent data suggest that the occurrence of immature T cells in the periphery of the immune system is extended during chronic phase in both mice and patients infected with *T. cruzi* [4], [27], [40].

The enhanced CD4^+^CD8^+^ cell homing to secondary lymphoid organs might be related to a selective expression of fibronectin receptors (VLA-4 and VLA-5) and also with TNF-receptors (TNF-R1 and TNF-R2). Accordingly, during *T. cruzi* infection, VLA-4 and VLA-5 are enhanced in CD4^+^CD8^+^ cells [4]. As ascertained by cell sorting of thymic subpopulations followed by qPCR, 14 days after infection, animals also exhibited enhanced expression levels of both mRNA TNF-α receptors in the CD4^+^CD8^+^ subpopulation, when we observed an increased export of this subset modulated by intrathymic TNF-α contents. Moreover, the relative amount of mRNA TNF-R2 seems to be higher than the TNF-R1 in these cells. This is in keeping with the putative role of TNF-R2 in cell migration events [36], [37], while TNF-R1 is believed to be more related to induction of apoptosis [28]–[30]. Overall, these data suggest that CD4^+^CD8^+^ cell migration may be favored by up-regulation of fibronectin and TNF-α receptors.

T cells are crucial in protective immunity against *T. cruzi*, but at the same time are involved in the autoimmune phenomenon seen in chronic Chagas disease [38], [39]. In this respect, our results reinforce the idea that the autoimmune component in Chagas disease may be partly related to the intrathymic migratory abnormalities with consequent export of immature cells. In this context, we unravelled herein the role of TNF-α mediated interactions in this overall process. Yet, the precise contribution of immature thymocytes to the immunopathological events occurring in Chagas disease deserves further investigation.
